# A Bayesian spatio-temporal model for forecasting *Anaplasma* species seroprevalence in domestic dogs within the contiguous United States

**DOI:** 10.1371/journal.pone.0182028

**Published:** 2017-07-24

**Authors:** Yan Liu, Stella C. Watson, Jenna R. Gettings, Robert B. Lund, Shila K. Nordone, Michael J. Yabsley, Christopher S. McMahan

**Affiliations:** 1 Department of Mathematical Sciences, Clemson University, Clemson, SC, United States of America; 2 Department of Molecular and Biomedical Sciences, Comparative Medicine Institute, North Carolina State University, College of Veterinary Medicine, Raleigh, NC, United States of America; 3 Southeastern Cooperative Wildlife Disease Study, Department of Population Health, College of Veterinary Medicine and the Warnell School of Forestry and Natural Resources, The University of Georgia, Athens, GA, United States of America; Johns Hopkins University, UNITED STATES

## Abstract

This paper forecasts the 2016 canine *Anaplasma* spp. seroprevalence in the United States from eight climate, geographic and societal factors. The forecast’s construction and an assessment of its performance are described. The forecast is based on a spatial-temporal conditional autoregressive model fitted to over 11 million *Anaplasma* spp. seroprevalence test results for dogs conducted in the 48 contiguous United States during 2011–2015. The forecast uses county-level data on eight predictive factors, including annual temperature, precipitation, relative humidity, county elevation, forestation coverage, surface water coverage, population density and median household income. Non-static factors are extrapolated into the forthcoming year with various statistical methods. The fitted model and factor extrapolations are used to estimate next year’s regional prevalence. The correlation between the observed and model-estimated county-by-county *Anaplasma* spp. seroprevalence for the five-year period 2011–2015 is 0.902, demonstrating reasonable model accuracy. The weighted correlation (accounting for different sample sizes) between 2015 observed and forecasted county-by-county *Anaplasma* spp. seroprevalence is 0.987, exhibiting that the proposed approach can be used to accurately forecast *Anaplasma* spp. seroprevalence. The forecast presented herein can *a priori* alert veterinarians to areas expected to see *Anaplasma* spp. seroprevalence beyond the accepted endemic range. The proposed methods may prove useful for forecasting other diseases.

## Introduction

Canine anaplasmosis is caused by gram-negative intracellular bacteria of the family Anaplasmataceae within the order Rickettsiales [1]. *Anaplasma* spp. bacteria are transmitted through the bite of infected ticks, with different tick species transmitting distinct types of *Anaplasma* bacteria in different regions of the country. *A. phagocytophilum* is transmitted by *Ixodes scapularis* and *I. pacificus* and maintained in a vector-reservoir-host system similar to that of *Borrelia burgdorferi* (the causative agent of Lyme disease), with the highest canine *A. phagocytophilum* seroprevalence reported in the Northeast, upper Midwest and along the west coast of California [2]. Although an important canine pathogen, *A. phagocytophilum* is also zoonotic and causes human disease in the same regions where Lyme disease occurs. In contrast, *A. platys* is presumed to be transmitted by *Rhipicephalus sanguineus*, and has relatively low prevalence across the contiguous United States with a slightly higher prevalence seen in the southern states [2]. Dogs in the southern U.S. (Florida, Georgia, North and South Carolina, Tennessee and Texas) show equivalent seroconversion to both *A phagocytophilum* and *A. platys* [2], suggesting exposure to multiple tick vectors. Veterinary wellness exams commonly include annual screening for exposure to *Anaplasma* spp., as well as *Ehrlichia* spp., *Borrelia burdgorferi* and *Dirofilaria immitis* (heartworm disease agent) using a rapid, in-house enzyme-linked immunosorbent assay (ELISA) platform (SNAP^®^4Dx^®^ and SNAP^®^4Dx^®^ Plus Test, IDEXX Laboratories, Inc., Westbrook, ME, USA) [3, 4]. These tests detect antibodies to both *A. phagocytophilum* and *A. platys* on a single spot and therefore no in-house speciation is possible. Of four million dogs tested for exposure to *Anaplasma* spp. in 2015, over 100,000 dogs were seropositive. Seroreactivity on these tests are interpreted by veterinary clinicians to indicate tick exposure and a history of transmission of *Anaplasma* spp.

Many, if not most, dogs remain asymptomatic following exposure to *Anaplasma* spp.. For example, in areas such as the northeastern US where disease is endemic, as many as 60% of dogs may have antibodies specific for *Anaplasma* spp. and the majority of these dogs do not have overt evidence of clinical disease [5, 6]. When symptomatic, dogs infected with *A. phagocytophilum* most commonly present with lethargy, fever and anorexia [7]. Thromobocytopenia is a hallmark of symptomatic *A. phagocytophilum* and *A. platys* infection [8–10], presumably because of platelet destruction [11]. Both splenomegaly and lymphadenopathy are reported and are thought to be associated with reactive lymphoid hyperplasia [11]; however it is critical to note that this is not specific to anaplasmosis as experimental infection of dogs with *Ehrlicia canis, E. chaffeensis, A. platys* and *A. phagocytophilum* results in similar histopathological lesions in lung, liver and spleen [12]. Finally, lameness due to neutrophilic polyarthritis, vomiting, diarrhea, neurologic abnormalities and epistaxis have been described [8–10, 13]. Because the majority of seropositive dogs are asymptomatic, current recommendations for veterinary care in *Anaplasma* spp. seroreactive dogs include a complete blood count with platelet count to determine if treatment is necessary [7].

Illnesses caused by tick-borne pathogens in animals and humans have increased over the last decade [14], due in part to the geographic expansion of tick populations beyond previously recognized endemic zones [15]. In a previous study, we evaluated potential explanatory factors for *Anaplasma* seroreactivity in dogs and and found seroprevalence increases with increasing precipitation and forestation coverage and decreases with increasing temperature, population density, relative humidity, and elevation [16]. Also, socioeconomic status and deer/vehicle collisions were positively and negatively correlated with canine *Anaplasma* spp. seroprevalence, respectively [16]. Given that many *Anaplasma* spp. infections are asymptomatic or mild, the relative distribution of disease risk is likely under-appreciated, particularly in non-endemic zones. As such, it would be advantageous to accurately forecast *Anaplasma* spp. seroprevalence on a local scale, providing an *a priori* alert to veterinarians in emerging areas of disease. Annual forecasts of emergent infection can inform veterinary and public health officials to shifting areas of infection, particularly in temperate regions of the US where *Anaplasma* spp. seroprevalence is generally absent, rare, or prevalence is highly influenced by annual variation in biotic or abiotic factors.

In the current study, we utilize the explanatory variables derived from our previous study to develop an *Anaplasma* spp. seroprevalence forecast model that uses Bayesian methods to account for the strong spatial and temporal dependencies that exist in *Anaplasma* spp. seroprevalence between counties and across time. Controlling for this autocorrelation, e.g. the similarity between observations as a function of time, provides us with estimates conducive to forecasting changes in the prevalence of exposure to *Anaplasma* spp. in space and time. The model described herein considers the climate, geographical, and societal factors included in the previous model, with the exception of deer/vehicle collisions, as potential predictors. We report on the fidelity of the forecast model by analyzing the relationship between predicted and actual *Anaplasma* spp. prevalence in 2015, and forecast the prevalence for 2016. Finally, we discuss the potential of canine *Anaplasma* seroprevalence to inform human practitioners, as human anaplasmosis is growing in recognition as a significant problem when present as a co-infection with *Borrelia burgdorferi* and is implicated in complicated, protracted Lyme disease [17].

## Materials and methods

### The data and baseline map construction

The data analyzed in this article consists of 11,437,537 *Anaplasma* spp. serology test results for dogs (obtained from IDEXX Laboratories, Inc. [18]). The tests were conducted in the contiguous United States from 2011 to 2015 and 3.21% were positive (i.e., 367,663 positive tests) for antibodies to *Anaplasma* spp. Along with the binary outcomes (positive or negative) the data also provides the county in which the test was conducted, and subsequently the analysis presented herein considers the county level aggregated totals; i.e., the number of positive and negative test results observed within each of the contiguous United States counties during the years of 2011 to 2015. As mentioned above, the tests are reported in the county in which the test was performed. No information is given on the travel or residence of the individual animal. In some cases, the county of testing may not be the county of exposure. There is also no testing history of the dogs or the reason why the tests were conducted, so repeat testing within the year may have occurred.

The explanatory factors considered here are those believed to be related to *Anaplasma* spp. seroprevalence in dogs, for which up to date data are available on a wide geographic scale. Table 1 presents the factors considered in our statistical analysis; i.e., climatic variables (annual temperature, precipitation, and relative humidity), geographic variables (county elevation, forestation coverage, and surface water coverage), and socio-economic variables (population density and median household income). For further details about these factors, such as their geographical distributions, please see [16]. Fine scale data on tick population levels is desirable for this analysis, but, to our knowledge, does not exist.

**Table 1:** Factors purported to be associated with *Anaplasma* spp. seroprevalence.

Factor	Data period	Scale	Notation	Range
Annual temperature (°F)	1895—2015	CD	*X*_*s*,1_(*t*)	[34.59, 77.14]
Annual precipitation (in)	1895—2015	CD	*X*_*s*,2_(*t*)	[0.30, 10.73]
Annual relative humidity (%)	2006—2015	CD	*X*_*s*,3_(*t*)	[17.98, 88.73]
Elevation (ft)	2012	C	*X*_*s*,4_(*t*)	[10, 14495]
Perc. forest coverage (%)	2012	C	*X*_*s*,5_(*t*)	[0.00, 32]
Perc. surface water coverage (%)	2010	C	*X*_*s*,6_(*t*)	[0.00, 91]
Population density (ppsm)	2011-2014	C	*X*_*s*,7_(*t*)	[0.10, 36041.11]
Median household income ($)	1997-2014	C	*X*_*s*,8_(*t*)	[20990, 125635]

For further discussion, including the source of each factor, see [19]. Note the following abbreviations are used: persons per square mile (ppsm), climate division (CD), county (C).

**Fig 1:**
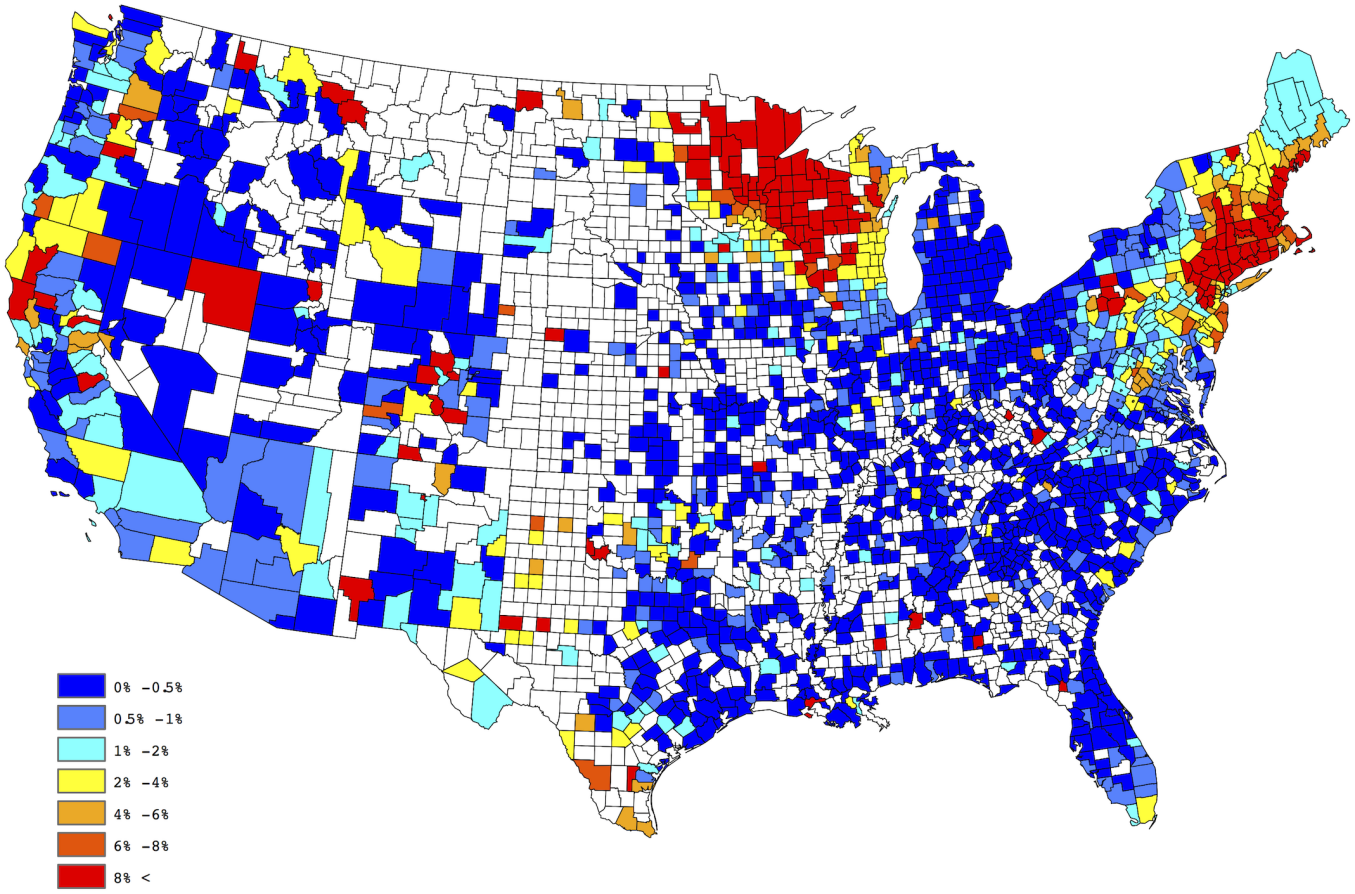
Empirical county-by-county *Anaplasma* spp. seroprevalence aggregated over 2011-2015.

Fig 1 provides a spatial depiction of the empirical *Anaplasma* spp. seroprevalence, after aggregating over the five years of available data. Here the empirical prevalence for each county is defined to be the number of positive tests divided by the total number of tests conducted. This figure tends to suggest a large degree of spatial correlation; i.e., empirical prevalences from counties close to each other tend to be similar. Moreover, within a given county the empirical prevalences also possess this property across time. These observations lead to the belief that both positive spatial and temporal correlation exists in our data. Thus, to offer a reliable evaluation of the putative factors, as well as to develop a predictive model, these effects must be taken into account. In the next section, a Bayesian regression model which acknowledges and accounts for both the spatial and temporal correlation is presented. It is worthwhile to note that some counties report relatively small number of test results, thus the interpretation of the empirical estimates depicted in Fig 1 may be slightly misleading, if one does not consider this effect. That is, a county reporting only 20 test results, with 1 being positive, results in an empirical estimate of 5%, whereas the same empirical estimate would be obtained for a county reporting 2000 test results, with 100 being positive. The salient point, more faith should be placed in the latter estimate, when compared to the former, since it is derived from a higher sample size.

**Fig 2:**
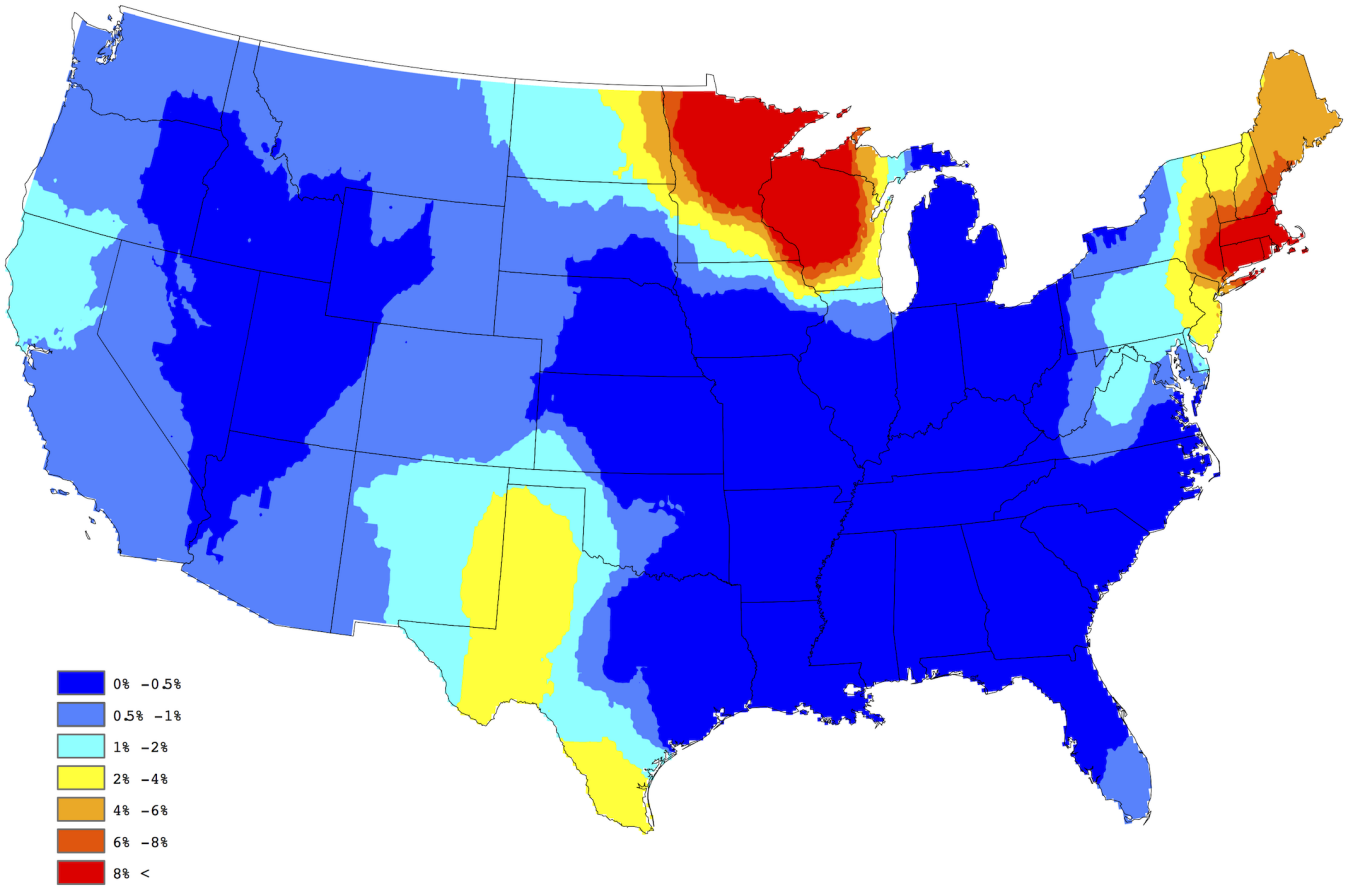
Baseline map of *Anaplasma* spp. seroprevalence.

In order to create a “baseline” map for *Anaplasma* spp. seroprevalence, the empirical estimates depicted in Fig 1 were further processed through a series of spatial smoothing techniques. First, a weighted head-banging algorithm was applied to the empirical prevalences. This procedure accomplishes two primary tasks; first, it acknowledges spatial correlation by forming a spatially oriented prevalence estimate through incorporating information from surrounding areas, and second, it down weights the influence of prevalence estimates which are derived from a relatively small number of tests. For example, in Colorado during 2015, 14,908 test results were reported, of which 112 were positive, translating to an observed prevalence of approx 0.75% for the state. Further, in Gunnison county, Colorado, only 3 test results were reported, of which 1 was positive, translating to an observed prevalence of 33%, which is obviously a small sample size issue. Weighted head banging directly acknowledges the available sample size from each county when computing the spatially oriented prevalence estimate. Weighted head banging was implemented using thirteen triples and the county level weights were chosen to be proportional to the number of county level observations which were reported over the five-year period; i.e., counties reporting more data were given more weight. In order to render a spatially complete map, Kriging (a common spatial interpolation technique) was used to interpolate prevalence estimates for counties not reporting data. This technique was implemented in ArcGIS using the default settings. Fig 2 provides our “baseline” map for *Anaplasma* spp. seroprevalence in domestic dogs within the contiguous United States. The “baseline” map presented in Fig 2 suggests two endemic zones of canine *Anaplasma* seroprevalence in the North-Central and Northeastern US, with lower but apparent seroprevalence in the Mid-Atlantic region, the Dakotas, and Western Texas.

### Model

This section outlines the model used to perform the spatio-temporal statistical analysis. As was previously alluded to, the available data exhibits both positive spatial and temporal correlation, and are measured on areal units (i.e., counties). Thus, to analyze these data, we employ a Bayesian hierarchical spatio-temporal regression model, where autocorrelated random effects are utilized to account for the spatial and temporal dependence. The use of Bayesian models for such analyses is omnipresent in similar application areas; for further discussion and a modern review see [20, 21].

For modeling purposes, the number of positive tests and total tests for county *s* at year *t* are denoted by *Y*_*s*_(*t*) and *n*_*s*_(*t*), respectively, where *s* ∈ {1, …, *S*} and *t* ∈ {1, …, *T*}. Following the proposals of [21–24], we relate the observed testing data to the factor information through the use of a Poisson regression model. Thus, the first level of our Bayesian hierarchical Poisson regression model is given by:

$$\begin{array}{c} Y_{s}\left(t\right)|n_{s}\left(t\right),p_{s}\left(t\right)∼\text{Poisson}\{n_{s}\left(t\right)p_{s}\left(t\right)\}, \end{array}$$ (1)

$$\begin{array}{c} \log\{p_{s}\left(t\right)\}=\beta_{0}+\sum_{k=1}^{8}\beta_{k}X_{s,k}\left(t\right)+\xi_{s}\left(t\right). \end{array}$$ (2)

In Eq (1), *p*_*s*_(*t*) denotes the *Anaplasma* spp. seroprevalence of county *s* at time *t*, ∼ means has the distributional type and the symbol ∣ indicates given quantity(ies). Proceeding with this notation, Eq (1) indicates that when *n*_*s*_(*t*) and *p*_*s*_(*t*) are known, the number of positive tests, the *Y*_*s*_(*t*), are conditionally independent across all the counties and they each follow a Poisson distribution with mean *n*_*s*_(*t*)*p*_*s*_(*t*). In Eq (2), log(⋅) denotes the natural logarithm, *X*_*s*,*k*_(*t*) is the *k*th factor measured on county *s* at time *t* (see Table 1), the *β*_*k*_ are regression coefficients, and *ξ*_*s*_(*t*) are spatio-temporal random effects used to account for the spatial and temporal dependence.

In order to capture spatial dependence, the proposed approach makes use of a conditional autoregressive (CAR) model. The CAR model was first proposed by [25], and has since seen numerous modifications and adaptations; e.g., see [26]. The version used here, which was adopted from [27], is now described. Let ***ϕ*** = (*ϕ*_1_, …, *ϕ*_*S*_)′ denote a random vector which follows a CAR model. Under our specification, one has that

$$\begin{array}{c} \phi ∼\text{N}(0,\tau^{2}{\left(D-\rho W\right)}^{-1}), \end{array}$$ (3)

where **W** = {*w*_*s*,*s*′_} is a *S* × *S* neighborhood adjacency matrix and **D** is a *S* × *S* diagonal matrix whose *s*th diagonal element denotes the number of counties bordering the *s*th county. The neighborhood adjacency matrix is constructed such that *w*_*s*,*s*′_ = 1 if the *s*th and *s*′th counties border one another, and *w*_*s*,*s*′_ = 0 otherwise. For notational convenience, we denote the relationship depicted in Eq (3) as ***ϕ*** ∼ CAR(*τ*^2^, *ρ*). Under this specification, it can be shown that the conditional distribution of *ϕ*_*s*_ is given by

$$\begin{array}{c} \phi_{s}|\phi_{-s},\tau^{2},\rho ,W∼\text{N}(\rho \frac{\sum_{s^{\prime }\neq s}w_{s,s^{\prime }}\phi_{s^{\prime }}}{\sum_{s^{\prime }\neq s}w_{s,s^{\prime }}},\;\;\frac{\tau^{2}}{\sum_{s^{\prime }\neq s}w_{s,s^{\prime }}}),\;\text{for}\;s=1,\ldots ,S, \end{array}$$ (4)

where ***ϕ***_−*s*_ = (*ϕ*_1_, …, *ϕ*_*s*−1_, *ϕ*_*s*+1_, …, *ϕ*_*S*_)′ is a vector of random effects for all counties except the *s*th one. In Eq (4), the conditional expectation of *ϕ*_*s*_, given its neighbors’ random effects, is the scaled (by *ρ* ∈ [0, 1]) average of the random effects of its neighbors. Therefore, *ρ* controls the spatial autocorrelation between bordering counties, with *ρ* = 0 indicating spatial independence and *ρ* close to 1 indicating strong spatial dependence. The parameter *τ*^2^ is a scaling variance parameter, and the conditional variance of *ϕ*_*s*_ is inversely proportional to the number of neighboring counties. Consequently, the conditional variance of *ϕ*_*s*_ is smaller (larger) than the conditional variance of *ϕ*_*s*′_ if the *s*th county has more (fewer) neighbors than the *s*′th county. This is reasonable since more neighbors relates to more information, and hence more certainty (i.e., a smaller variance).

Motivated by [22], our proposed model captures the spatial and temporal dependence through a multivariate autoregressive model of order one which is given by

$$\begin{array}{ccc} \xi_{1} & = & \phi_{1};\\ \xi_{t} & = & \phi \xi_{t-1}+\phi_{t},\;\text{for}\;t=2,\ldots ,T;\\ \phi_{t} & ∼ & \text{CAR}(\tau^{2},\rho ),\;\text{for}\;t=1,\ldots ,T, \end{array}$$ (5)

where, ***ξ***_*t*_ = (*ξ*_1_(*t*), …, *ξ*_*S*_(*t*))′ and ***ϕ***_*t*_ = (*ϕ*_1_(*t*), …, *ϕ*_*S*_(*t*))′ for *t* = 1, …, *T*. Note, in our proposed model the spatial random effects ***ϕ***_*t*_ are independent and identically distributed according to the CAR model described in Eq (3). The parameter *φ* controls the temporal correlation between consecutive years and lies within (−1, 1), this ensures a causal and stationary solution to the time series model (see [28]). Moreover, in the proposed model *φ* = 0 indicates temporal independence, while *φ* close to 1 indicates strong positive temporal correlation.

To complete the Bayesian model, the following prior distributions were specified:

$$\begin{array}{c} \beta_{k}∼\text{N}\left(0,1000\right),\;\text{for}\;k=0,\ldots ,8; \end{array}$$ (6)

$$\begin{array}{c} \varphi ∼\text{Uniform}(-1,1); \end{array}$$ (7)

$$\begin{array}{c} \rho ∼\text{Uniform}(0,1); \end{array}$$ (8)

$$\begin{array}{c} \tau^{-2}∼\text{Gamma}\left(0.5,0.05\right). \end{array}$$ (9)

Note, the prior distributions placed on the *β*_*k*_ are independent and weakly informative so that estimation and inference for these parameters are based mainly on the data. Priors for *φ* and *ρ* are uninformative, and were chosen for the same reason. For ease of computation, the prior for *τ*^−2^ was chosen due to conjugacy (the posterior and prior distributions are from the same distributional family). A Markov chain Monte Carlo (MCMC) posterior sampling algorithm for the model parameters and random effects was constructed, which made use of a combination of Gibbs and Metropolis-Hastings sampling steps [29]. In the algorithm, the response variable (i.e., *Y*_*s*_(*t*)) associated with counties not reporting data were treated as missing data and were subsequently sampled along with the other model parameters. The MCMC sampling algorithm was written and implemented in **C++** and **R**.

## Results

### Model assessment

A primary goal of this work was to evaluate the putative factors (see Table 1) with respect to their association with *Anaplasma* spp. seroprevalence within domestic canines in the contiguous United States. To accomplish this task, a full model with all eight factors was fitted to the data. From this fit, point estimates (means of posterior samples) and 95% highest posterior density (HPD) credible intervals were obtained for all regression coefficients; for further discussion and details about HPD intervals see [29]. Table 2 provides the point estimates and HPD intervals for each of the eight regression coefficients.

**Table 2:** Parameter estimates from the full model.

Factor	Estimate	95% HPD Interval
Annual temperature (°F)	-0.021	[-0.036, -0.008]
Annual precipitation (in)	-0.004	[-0.053, 0.037]
Annual relative humidity (%)	-0.001	[-0.008, 0.004]
Elevation (ft)	0.032	[0.002, 0.061]
Percentage forest coverage (%)	3.039	[1.914, 4.045]
Percentage surface water coverage (%)	0.398	[0.130, 0.692]
Population density (ppsm)	-2.765e-5	[-4.473e-5, -0.976e-5]
Median household income ($)	0.002	[-0.001, 0.005]

From Table 2, one can ascertain that 3 of the considered factors were insignificant in the full model at the considered significance level; i.e., the 95% HPD intervals for the regression coefficients associated with annual precipitation, annual relative humidity, and median household income contain zero. In order to develop a parsimonious model that only contains significant factors, 7 reduced models were fit to the data, each containing a different combination of the 3 questionable explanatory factors. In each of these additional model fits, these 3 factors were again deemed to be insignificant. Therefore, our selected model makes use of 5 explanatory factors; i.e., annual temperature, percentage forest coverage, percentage surface water coverage, elevation, and population density. Table 3 provides the point estimates of the regression coefficients along with 95% HPD intervals for the factors belonging to the selected model. In addition, the point estimates of the other model parameters are *φ* = 0.935, *ρ* = 0.999, and *τ*^2^ = 0.639, which reaffirms the assertion that strong positive spatial and temporal correlation are present in the data.

**Table 3:** Parameter estimates from the selected model.

Factor	Estimate	95% HPD Interval
Annual temperature (°F)	-0.019	[-0.031, -0.004]
Percentage forest coverage (%)	2.881	[1.855, 4.065]
Percentage surface water coverage (%)	0.389	[0.112, 0.675]
Elevation (ft)	0.033	[0.005, 0.058]
Population density (ppsm)	-3.090e-5	[-4.635e-5, -1.464e-5]

**Fig 3:**
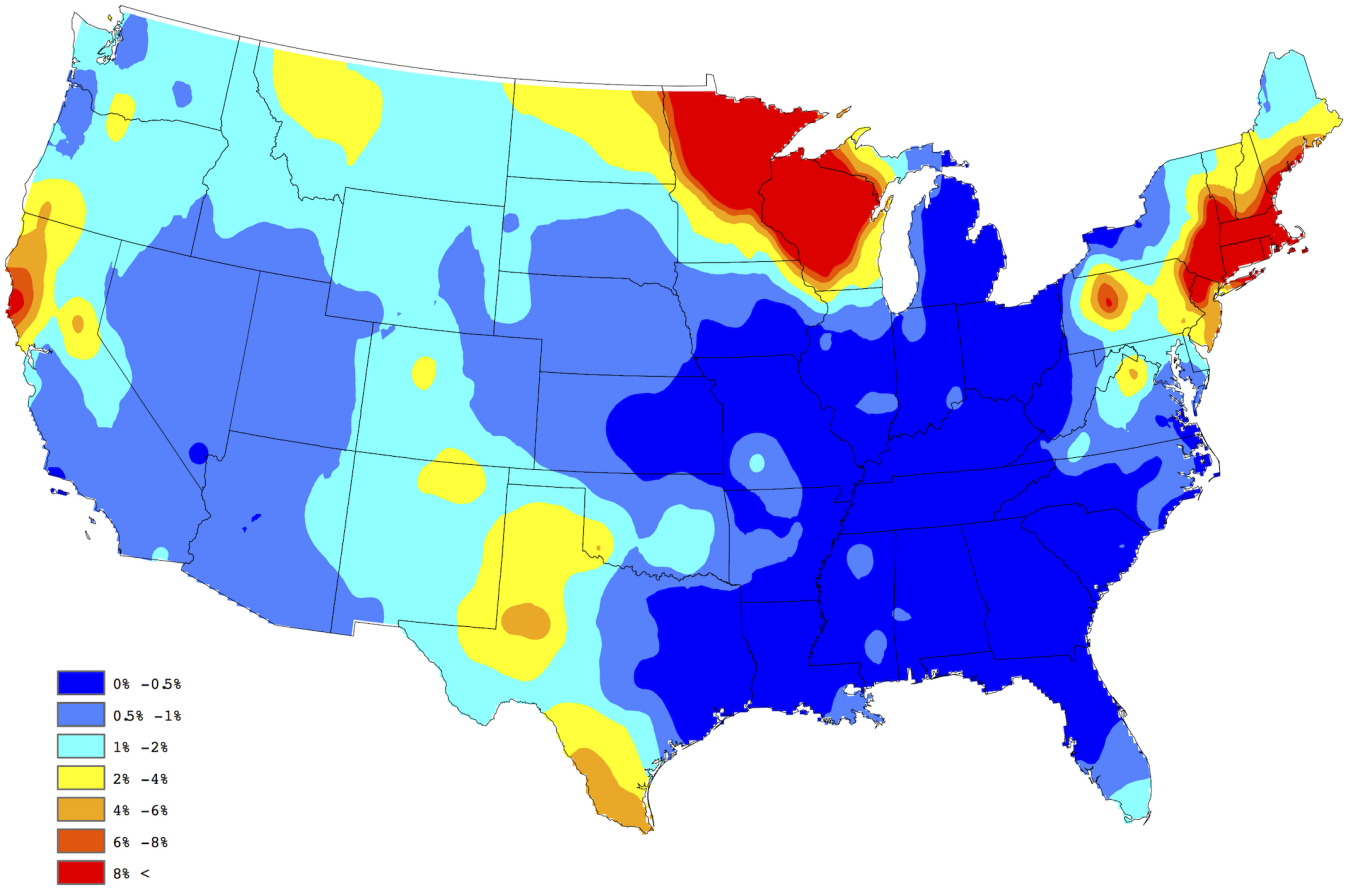
Aggregated model-based estimates of *Anaplasma* spp. seroprevalence.

In order to assess the adequacy of our selected model, a figure analogous to Fig 2 was created using the model based estimates of the county level prevalences. In particular, our fitted model provides a yearly prevalence estimate for each county, regardless of whether the county reports data. Thus, to obtain a single prevalence estimate for each county, we averaged over the 5 yearly estimates available from the model fit; i.e., our aggregated estimate for the *s*th county is given by ${\hat{p}}_{s}=5^{-1}\sum_{t=1}^{5}{\hat{p}}_{s}\left(t\right)$, where ${\hat{p}}_{s}\left(t\right)$ is the prevalence estimate resulting from the selected model for the *s*th county during the *t*th year. Fig 3 provides a depiction of these results, after Kriging (again default settings were used in ArcGIS). By comparing these two figures one will note that the proposed Bayesian spatio-temporal model appears to provide a good fit to these data. In fact, the correlation between the sets of estimates depicted in Figs 2 and 3 is 0.902, thus confirming that a strong agreement exists between the empirical and model based estimates. Note, this summary measure did not consider estimates from counties not reporting data.

### Forecasting

In this section we provide details on how the model developed in the previous section can be utilized to construct a forecast of future trends of *Anaplasma* spp. seroprevalence across the contiguous United States. Essentially, our strategy relies on forecasting the value of the 5 significant explanatory factors as well as the spatio-temporal random effects for the upcoming year. Since forestation, water coverage, and elevation are relatively stable over time, the forecasted values of these factors are simply taken to be their most recent observations. In order to forecast annual temperature and population density, we make use of the same techniques outlined in [30].

The spatio-temporal random effects for the next year were predicted using the relationship depicted in Eq (5). In particular, for each value of *τ*^2^, *ρ*, *φ* and ***ξ***_*t*_ which are available from the posterior sample, we obtain a predicted value of the spatio-temporal random effect for the next year as ***ξ***_*t*+1_ = *φ* ***ξ***_*t*_ + ***ϕ***_*t*+1_, where ***ϕ***_*t*+1_ was randomly generated from a N(**0**, *τ*^2^(**D** − *ρ* **W**)^−1^). This process generates posterior predictive samples (see [29] for additional details) of ***ξ***_*t*+1_, which can then be used to forecast next year’s *Anaplasma* spp. seroprevalence.

**Fig 4:**
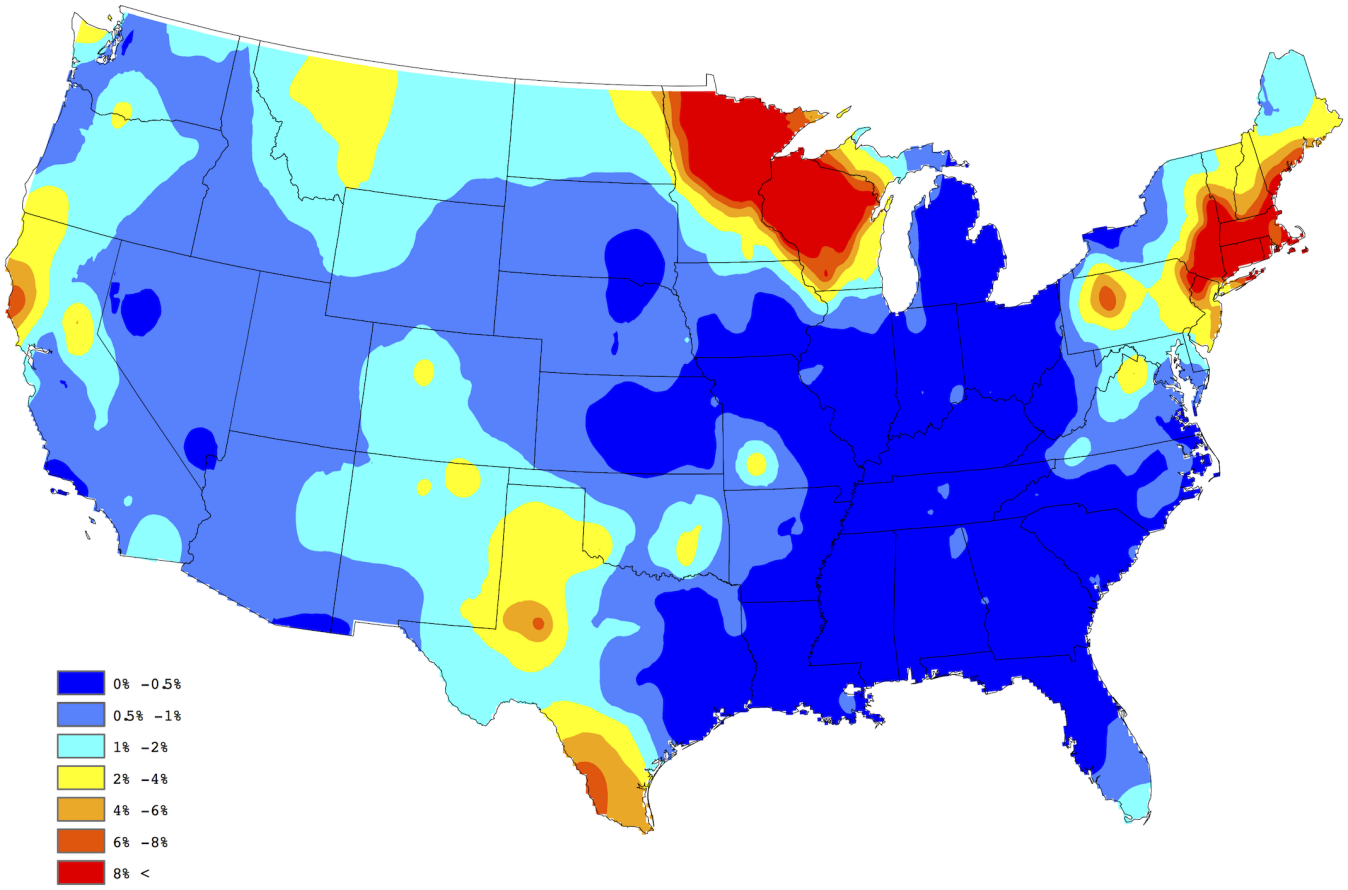
2015 forecasted *Anaplasma* spp. seroprevalence.

**Fig 5:**
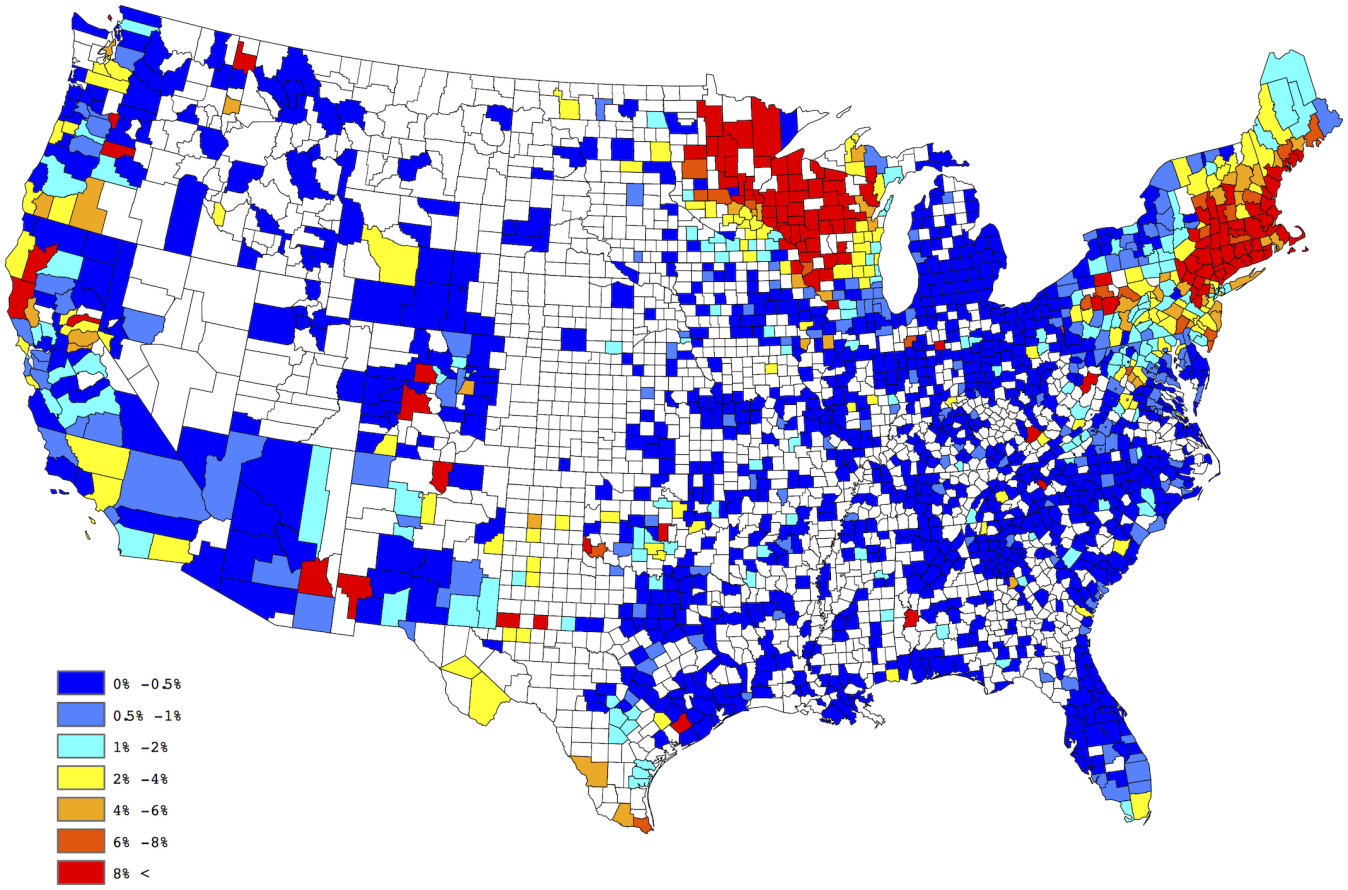
2015 observed *Anaplasma* spp. seroprevalence.

**Fig 6:**
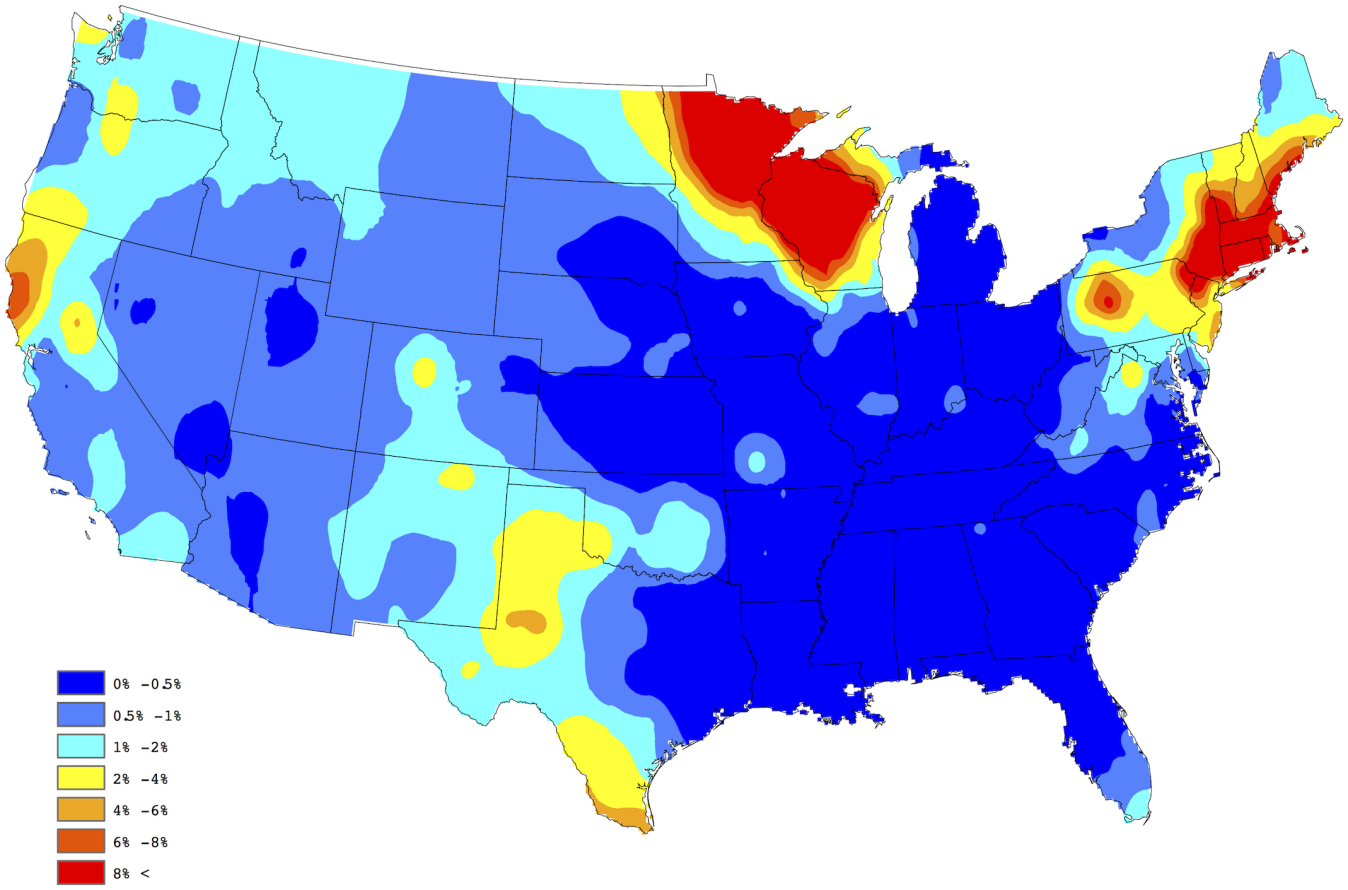
2016 forecasted *Anaplasma* spp. seroprevalence.

In order to assess the fidelity of the proposed forecasting procedure, the 2015 test and factor data were removed, and our approach was used to forecast the 2015 county level prevalences using the 2011-2014 test and factor data only. Figs 4 and 5 present the forecasted and observed prevalences during 2015, respectively. The weighted correlation between the forecasted and observed prevalences (for counties reporting data in 2015), is 0.987, demonstrating that *Anaplasma* spp. seroprevalence can be accurately forecasted through the proposed approach. Here, a weighted correlation, with weights being set to be equal to *n*_*s*_(*t*), is used to account for different sample sizes within each county, for further discussion see [30]. Fig 6 provides our 2016 forecast of canine *Anaplasma* spp. seroprevalence within the contiguous United States.

## Conclusion and discussion

In this study, we present for the first time a fully Bayesian approach to forecasting canine *Anaplasma* spp. seroprevalence in the absence of detailed information on the distribution and abundance of the two primary vectors of disease: *Ixodes* spp, and *Rhipicephalus sanguineus* and pathogen prevalence in vectors. Surveillance of these medically important vectors remains of high importance; however, annual analysis of vector distribution and molecular characterization of pathogen burden will remain economically and logistically unfeasible in many areas in the face of changing climate and habitats. As such, we have developed a model for forecasting spatial and temporal patterns of risk of exposure to *Anaplasma* spp. based on canine seroprevalence data. Data in our study are limited by lack of detailed *Anaplasma* species specificity; however, from the recent species-level serologic analysis conducted by Qurollo et al., we can infer that the majority of observed *Anaplasma* spp. seroprevalence in the Northeast, upper Midwest and west coast of California is likely the result of exposure to *A. phagocytophilum*, and seroprevalence in the southern and western US due to exposure to either *A. platys* or *A phagocytophilum* [2]. In California, there is a higher liklihood of the antibodies being due to *A phagocytophilum* exposure whereas positives in Texas and other southeastern states is due to *A. platys* exposure.

A major strength of our surveillance effort is that *Anaplasma* spp. seroprevalence data are acquired on a monthly basis [31], providing a robust and timely source of information about the dynamic change of *Anaplasma* spp. exposure across the contiguous US. These data hold strong promise for longitudinal studies to best understand the dynamic nature of *Anaplasma* spp. prevalence over time. For veterinary healthcare practitioners specifically, this information is of critical importance as disease is often mild or unapparent in the dog, requiring both a working knowledge of risk paired with an impetus to pursue further diagnostics to determine when treatment is necessary for the health of the dog. Similarly, a deeper understanding of risk enhances educational opportunities, with the potential to prevent the indiscriminate use of antibiotics [32] in asymptomatic dogs without thrombocytopenia or other significant blood count changes. Finally, even if dogs with *Anaplasma* spp. are asympotomatic, this exposure indicates tick infestation which may have or will lead to infection with other tick borne pathogens that may need to be considered.

The forecast model uses 5 years of historical data acquired from IDEXX Laboratories, Inc and previously published relevant covariate factors [16] to help explain variability in our aggregated dataset and to strengthen inferences from our Bayesian spatio-temporal model. As previously described in our explanatory model of *Anaplasma* spp. seroprevalence, the prevalence of seropositivity increases with increasing precipitation and forestation coverage and decreases with increasing temperature, population density, relative humidity, and elevation. Socioeconomic status and deer/vehicle collisions were positively and negatively correlated with canine *Anaplasma* seroprevalence, respectively [16]. The potential drivers of infection in the current model vary some from the previously published model because of the differences in statistical methods used. For forecasting, a Bayesian model was chosen because of the ability to control for confounding caused by spatial and temporal autocorrelation seen in disease prevalence data. The associations of annual temperature, forest coverage, and population density remain similar between our previous explanatory model and our forecast model. In contrast, percentage of water coverage was significant in a Bayesian model, but not in the explanatory model [16]. Elevation was found to have a positive association in a Bayesian forecast model, whereas the association was negative in the explanatory model. This can be explained in part by the distribution of the two *Anaplasma* spp. of dogs. *Anaplasma phagocytophilum* is the primary cause of seropositivity in dogs in many regions of the United States, and this pathogen is found in high prevalences in the northeastern United States, a region that is higher in elevation and lower temperature, compared to the non-endemic regions of the South and southeastern United States. We acknowledge a limitation in our seroprevalence dataset that it is a “presence-absence” model presented at a crude spatial scale, and as such covariates such as elevation are perceived as uniform over large geographical areas. By extending our model to include climate variables over a similarly large spatial area it was revealed that the association at the regional level is related to the climatic conditions. Despite the aformentioned limitations, using data from 2011-2014 we evaluated observed versus forecasted *Anaplasma* spp. seroprevalence in 2015, resulting in a weighted correlation between the two maps of 0.987. Given the striking fidelity of our forecast model, we report our 2016 forecast for canine *Anaplasma* seroprevalence in the contiguous United States.

Using our described methodology, canine *Anaplasma* spp. seroprevalence is forecasted to be high in regions of the US endemic for *Ixodes scapularis*: the Mid-central and Northeastern states of Connecticut, Delaware, Maine, Massachusetts, Maryland, Minnesota, New Hampshire, New Jersey, New York, Pennsylvania, Rhode Island, Vermont and Wisconsin. Six of these states: New York, Connecticut, New Jersey, Rhode Island, Minnesota, and Wisconsin, account for 90% of all reported cases of human anaplasmosis due to infection with *A. phagocytophilum* [33]. Beyond these endemic boundries, we observed moderate to high frequency of canine *Anaplasma* spp. seroprevalence in Northern California, North Dakota and Texas. Our forecast of canine *Anaplasma* spp. seroprevalence in Texas is notable given a recent study by Movilla et al. who documented, for the first time, canine seroprevalence of *Anaplasma* spp. in several states of Mexico [34]. The authors reported the highest seroprevalence in northwestern Mexico (16.4%), with the lowest in the north-central states of the country (0.6%). Wildlife species in Texas have been documented to harbor *A. phagocytophilum* as well [35]. It is well established that incursions by humans into natural habitats make the boundary between wildlife, humans and domestic animals more permeable, and thereby make the spillover of vector-borne disease more likely. Collectively, the presence of *Anaplasma* spp. in both domestic dogs in Mexico and wildlife in the south-central US indicate a greater need for annual testing of dogs for *Anaplasma* spp. in southern US border states. Similarly, evidence of canine *Anaplasma* spp. seroprevalence in Northern California and along the Canadian border suggests annual testing for *Anaplasma* spp. in these regions during veterinary wellness visits is strongly advised.

Human infection with *A. phagocytophilum* has been reported in non-endemic regions of the United States, including southeastern and south-central states [33, 36], where high levels of *Ixodes* spp. transmitting *A. phagocytophilum* have yet to be documented. As such, there remains some controversy regarding true establishment of human anaplasmosis outside of endemic states [33, 36]. The Centers for Disease Control and Prevention (CDC) suggests some of these human cases may be due to patient travel to states with higher levels of disease, or the misdiagnosis of anaplasmosis in patients actually infected with another tick-borne disease, such as ehrlichiosis or Rocky Mountain Spotted Fever. Our canine seroprevalence data indicate that *Anaplasma* spp. are being transmitted to dogs in Northern Texas and Northern California, as well as along the Canadian border in North Dakota and Montana, although the species is unknown. While canine seroprevalence for *B. burgdorferi* is an established surveillance tool for human Lyme disease [37, 38], it remains to be determined whether canine *Anaplasma* seroprevalence can provide a similar risk assessment tool for human epidemiologists. Regardless, as *A. phagocytophilum* is a potential zoonotic pathogen, it is important to be aware that these organisms are enzootic in non-endemic regions of the United States and, and in particular, in the regions bordering the south-central US. Finally, it is important to note that while canine Lyme disease caused by *Borrelia burgdorferi* and transmitted by *Ixodes* spp. is now increasing in the Great Lakes region of the US as *Ixodes* ticks converge from the Mid-Central state and Northeastern states [15], a similar pattern of elevated *Anaplasma* spp. seroprevalence was not evident in our current dataset. It is unclear whether *Ixodes scapularis* ticks in this region do not harbor *Anaplasma* spp. or whether canine test data are currently too sparse in this region to detect notable increases in seroprevalence.

While canine anaplasmosis is most often asymptomatic and self-limiting, there is growing recognition in human and veterinary medicine that *Anaplasma* spp. may represent a significant health threat as a co-morbidity. Indeed, co-infection with *Ixodes*-borne pathogens is prevalent and increasingly problematic worldwide [39]. In humans, most co-infections involve two of the three major human pathogens, *B. burgdorferi* sensu lato, *A. phagocytophilum*, and *Babesia* spp. Such co-infections have been documented to occur in up to 28% of *I. scapularis* ticks in Lyme disease-endemic areas in the US [40]. Co-infection with multiple tick-borne pathogens can increase Lyme disease severity and has been attributed to long-term sequela in patients. Human patients with these concurrent illnesses experienced a greater number of symptoms for a longer duration than patients with Lyme disease alone [41, 42]. Co-exposure of dogs to *B.burgdorferi* and *A. phagocytophilum* has been reported, with co-exposure rates greatest in the Northeast (6.2%) and Mid-Atlantic region (1.8%) of the US [2]. Veterinary patient outcomes in the presence of co-infection are not as thoroughly defined; however, clinically ill dogs that are seropositive for *B.burgdorferi* and *A. phagocytophilum* have been noted to be twice as likely to have lameness, joint pain, and joint effusion than dogs with single infections [5, 13]. In general, veterinary clinicians are increasingly concerned co-infections complicate interpretation of clinical manifestations of disease and therefore potentially confound treatment [2]. As such, in *Ixodes* endemic regions annual screening for multiple vector-borne pathogens is strongly warranted. Beyond annual screening, year-round use of acaracides in dogs can reduce tick infestation, thereby reducing the potential for tick-borne pathogen transmission [43, 44].

There are a few limitations to this study that need to be addressed. As mentioned above, samples are submitted by veterinary clinics, indicating that this population of dogs are under the care of a veterinarian. This suggests that these data are representative of a subpopulation of dogs that are more likely to receive tick preventatives and thus more protected from exposure to ticks. Therefore, prevalence estimates presented here are likely to be conservative. A second limitation to note is the county of testing. For a small sample of dogs, the county of testing may not reflect the county of residence, and may also misclassify the county of exposure for dogs exposed while traveling. Unfortunately, counties of residence and travel history are not known for these data, so interpretations are made with these limitations in mind.

In summary, we have forecasted the distribution of the canine seroprevalence at the national level with available canine serology data and easily accessible climate, geographical, and societal factors. The high degree of fidelity between actual vs. forecasted seroprevalence in this model and ease of data input provides veterinary and public health officials with an invaluable tool for informing emerging risk of exposure to *Anaplasma* spp. disease potential, and importantly the possibility of risk of co-infection with other tick-borne disease. Information should be shared with dog owners to better facilitate appropriate preventative care of healthy animals and diagnosis and treatment of ill patients. As we continue to learn more about the association between the distributions of anaplasmosis in humans and dogs, this forecasting model may become useful for public health practitioners as well as a critical source of information on the ecology and changing distribution of *Anaplasma* spp.
